# Supplementary material for: Cell Membrane-Integrated Neuroligin-1 Regulates the Anti-Inflammatory Effects of CRC Cell-Derived Exosomes
Source: Int J Mol Sci. 2025 Jan 9;26(2):503. doi: 10.3390/ijms26020503 (PMC11765187; doi:10.3390/ijms26020503)
Supplement: Supplementary file 1 [file ijms-26-00503-s001.zip › ijms-3416468-supplementary.pdf]

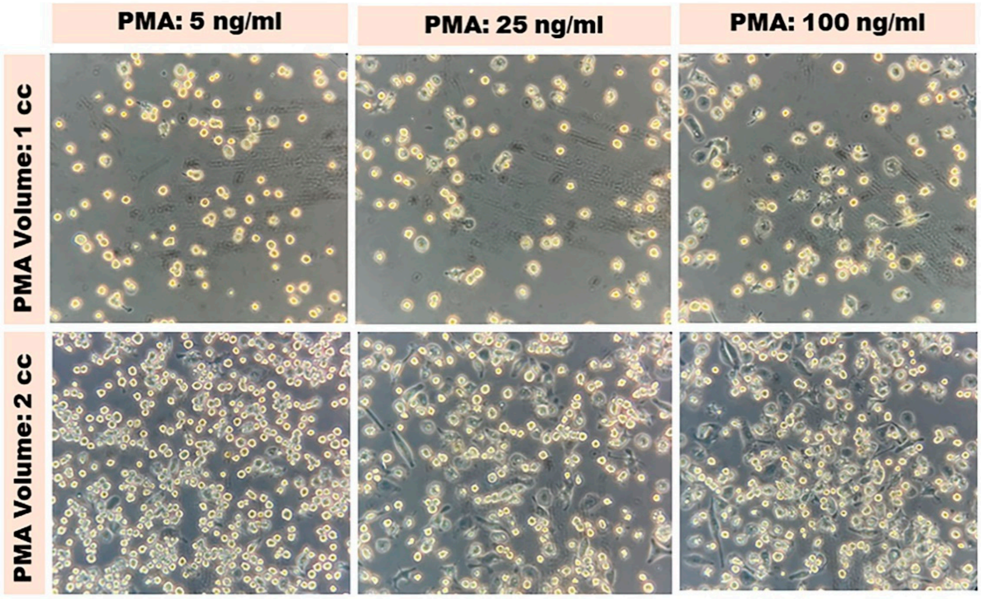

Figure S1. The effect of the PMA concentration and volume on the attachment density of THP-1macrophages; THP-1 monocyte cells (500,000 cells/well) were treated with various concentrations (5, 25, and 100 ng/mL) and volumes (1 vs. 2 mL) of the PMA to induce M0 macrophage polarization in a 6-well cell culture plate. The magnification scale of the images is 10X.

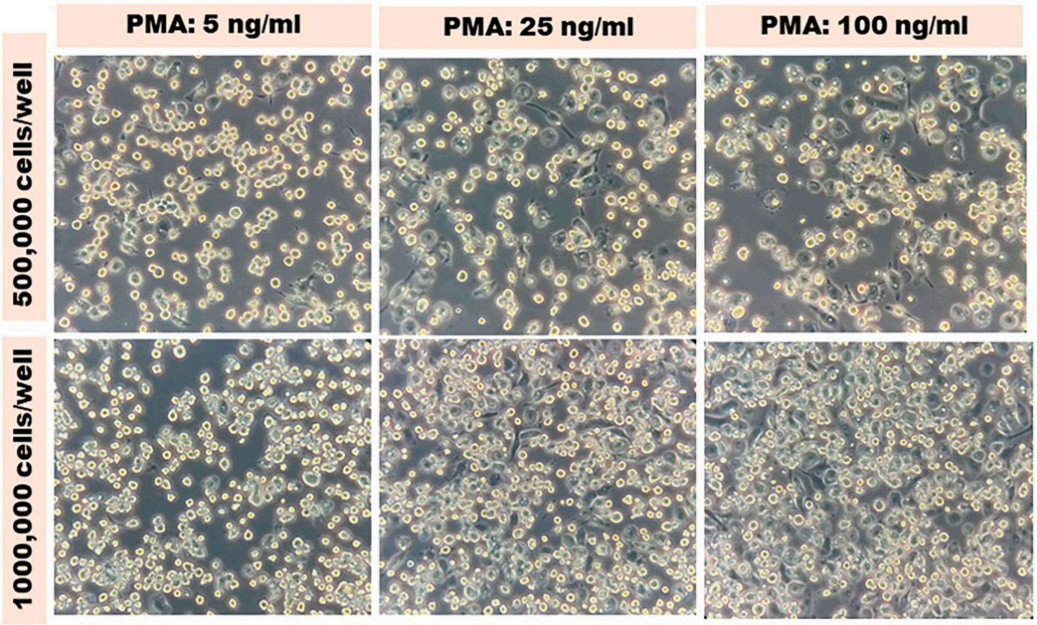

Figure S2. The effect of the seeding density of the THP-1 monocyte cells on the attachment density of THP1-macrophages; THP-1 monocyte cells (500,000 cells/well vs. 1000,000 cells/well) were treated with different 2 mL of various concentrations of the PMA (5, 25, and 100 ng/mL) to induce M0 macrophage polarization in 6-well cell culture plate. The magnification scale of the images is 10X.
